# The effect of cognitive-behavioral therapy and sexual health education on sexual assertiveness of newly married women

**DOI:** 10.1186/s12888-023-04708-w

**Published:** 2023-03-28

**Authors:** Sanaz Jangi, Roghaiyeh Nourizadeh, Niloufar Sattarzadeh-Jahdi, Mahmoud Farvareshi, Esmat Mehrabi

**Affiliations:** 1 Student Research Committee, Midwifery Department, Tabriz University of Medical Sciences, Tabriz, Iran; 2 Midwifery Department, Faculty of Nursing and Midwifery, Tabriz University of Medical Sciences, Tabriz, Iran; 3 Razi Psychiatric Hospital, Tabriz University of Medical Sciences, Tabriz, Iran

**Keywords:** Cognitive-behavioral therapy, Sex education, Sexual assertiveness, Sexual satisfaction, Newly married women

## Abstract

**Background:**

The present study aimed at investigating the effect of sexual health education and cognitive-behavioral therapy (CBT) on sexual assertiveness (primary outcome) and sexual satisfaction (secondary outcome) of newly married women.

**Method:**

This RCT was conducted on 66 newly married women with cases in pre-marriage counseling centers in Tabriz, Iran. Participants were assigned into three groups using block randomization. Eight group sessions of CBT were held for one of the intervention groups (*n* = 22) and 5–7 sessions of sexual health education for other intervention group (*n* = 22). The control group (*n* = 22) received neither education nor counseling during the research. The data were collected using the demographic and obstetric characteristics, Hulbert sexual assertiveness index, and Larson sexual satisfaction questionnaires, and analyzed using ANOVA and ANCOVA tests.

**Results:**

The mean (standard deviation: SD) score of the sexual assertiveness and sexual satisfaction in the CBT group enhanced from 48.77 (13.94) and 73.13 (13.53) before the intervention to 69.37 (7.28) and 86.57 (7.5) after the intervention, respectively. The mean (SD) score of the sexual assertiveness and sexual satisfaction in the sexual health education group increased from 48.9(11.39) and 74.95 (8.30) before the intervention to 66. 94 (7.42) and 84.93 (6.34) after the intervention, respectively. The mean (SD) score of the sexual assertiveness and sexual satisfaction in the control group changed from 45.04 (15.87) and 69.04 (10.75) before the intervention to 42.74 (14.11) and 66.44 (10.11) after the intervention, respectively. Eight weeks after the intervention, the mean scores of sexual assertiveness and sexual satisfaction in two intervention groups were more than that in the control group (*P* < 0.001), However, there was no significant difference between the two intervention groups (*P* > 0.05).

**Conclusion:**

The results of this research indicated that CBT and sexual health education are effective in improving women’s sexual assertiveness and sexual satisfaction. Considering that sexual health education, does not require complex counseling skills compared to CBT, it can be used as a preferred intervention in promoting sexual assertiveness and satisfaction of newly married women.

**Trial registration:**

Iranian Registry of Clinical Trials: IRCT20170506033834N8. Date of registration: 11.09.2021. URL: http://en.irct.ir.

## Background

At the beginning of a new married life, women tend to keep their husbands satisfied. Therefore, they prioritize their husbands’ sexual needs and refrain from expressing their sexual needs and desires, which consequently results in women’s sexual dissatisfaction [1]. The lack of correct information, weak communication and sexual skills, and unrealistic expectations in marital relationships play a significant role in the occurrence of disagreement and reduction of sexual satisfaction [2]. Therefore, sexual assertiveness is considered as one of the most important factors in sexual satisfaction.

The term sexual assertiveness is defined as the cognitive, behavioral, and emotional aspects of talking and communicating with a partner about sexual needs. This concept includes the ability to self-disclose and initiate or refuse sexual intercourse [3]. In most societies and cultures, women have problems with their sexual assertiveness, as it is difficult for them to express their needs and maintain their individual independence in the marital relationship [4]. Based on the results of the study of Jennifer, talking about sexual issues and women’s sexual assertiveness enhances by increasing the length of marriage [5]. Evidence shows that women with more sexual expression power experience higher sexual satisfaction [6, 7].

Sexual satisfaction, as an important component of sexual health and outcome of sexual well-being, is effective in creating and maintaining a happy marital relationship [8]. Sexual satisfaction is affected by both mental and physical dimensions [9]. Various factors, such as sexual knowledge and attitude, frequency of sexual intercourse, and experience of orgasm affect the level of sexual satisfaction [10]. The sexual dissatisfaction may cause a feeling of failure and insecurity in the sexual relationship [11].

Many educational and psychological interventions have been used to solve sexual problems and improve marital conflicts [12–16]. Obviously, achieving the sexual and reproductive health is influenced by literacy, knowledge, and awareness of various aspects of this field, as well as the appropriate self-care practices of the individual [17]. In fact, adequate sexual health literacy increases a person’s skill in analyzing, judging, discussing, making decision, and changing sexual behavior and empowers him/her in providing, maintaining, and improving sexual health [18]. Ranjbaran et al. reported that education and counseling significantly improve sexual health in adolescents [19].

Cognitive-behavioral therapy (CBT) is regarded as one of the psychological interventions conducted in the field of promoting sexual assertiveness and satisfaction. The main basis of CBT is to focus on thoughts and perceptions and their impact on emotions and behavior [20]. The CBT helps clients with sexual problems through correcting sexual misconceptions, practicing sexual self-expression, having shared responsibility in sexual matters, being able to solve problems, eliminating anxiety in sexual relations, training communication skills and sexual techniques, making changes in sexual behavior and cognitive reconstruction [21]. Chizary et al. reported that Assertiveness-Focused CBT improved sexual function among adults [22]. Fuhrer, Weinhardt et al. found that CBT approach was effective in increasing sexual assertiveness of women who were at high risk for human immunodeficiency virus (HIV) [23].

Considering that the early years of marriage may be associated with women’s inability in sexual assertiveness and sexual expression, which subsequently leads to the sexual dissatisfaction, and due to the lack of the comparison of the effect of educational and psychological interventions on sexual assertiveness and satisfaction in previous studies, the present study aimed at investigating the effect of sexual health education and CBT on sexual assertiveness (primary outcome) and sexual satisfaction (secondary outcome) of newly married women. Investigating the most effective intervention on sexual satisfaction in women with low sexual assertiveness is a matter of great importance.

## Study hypotheses

1. The mean score of sexual assertiveness is different between three groups.
2. The mean score of sexual satisfaction is different between three groups.

## Method

### Study design and participants

This randomized controlled clinical trial was conducted on 66 newly married women with cases in Asad-Abadi and Haft-e-Tir pre-marriage counseling centers in Tabriz. All Iranian couples previously referred to pre-marital centers for pre-marriage tests were selected as sample.

The inclusion criteria were newly married women (1—3 years passed from their marriage), scoring less than 50 in the Hulbert Sexual Assertiveness Index, having at least elementary education, first marriage, and living in a shared house. The exclusion criteria included aged below 18-year, pregnant or breastfeeding women, being candidates for divorce, history of mental disorders, such as depression, suffering from physical diseases, infertility, forced marriage, and addiction of husband or wife.

The sample size was calculated using G-Power software. According to the study of Parva et al.[24] based on the variable of sexual assertiveness and considering M_1_ = 46.21, with the assumption of 20% increase in the mean score of sexual assertiveness after the intervention (M_2_ = 55.45), SD_1_ = SD_2_ = 10.43, two-sided α = 0.05, and Power = 80%, sample size was obtained 22 in each group. Further, according to the study of Mofid et al. [25] based on the variable of sexual satisfaction and regarding M_1_ = 92.9, assuming 15% increase in the mean score of sexual satisfaction following the intervention (M_2_ = 106.8), SD_1_ = SD_2_ = 9.6, two-sided α = 0.05, and Power = 90%, the sample size was estimated 12 in each group. Therefore, total sample size was considered 66 by taking into account the larger sample size for each group (*n* = 22).

### Sampling

After registering on the website of the Iranian Registry of Clinical Trials (IRCT20170506033834N8), sampling was done in Asad-Abadi and Haft-e-Tir marriage counseling centers in Tabriz from June to March 2021. The researcher (first author) attended the marriage counseling centers, identified eligible women using the archived information, and after calling and explaining the research objectives, invited those who wanted to participate in the study to attend marriage counseling centers at the appointed time. In the face-to-face session, after completing the questionnaires, women who scored less than 50 in the Hulbert sexual assertiveness index and less than 76 in Larson sexual satisfaction questionnaire were explained the method of the study. Then, they completed the written informed consent form.

### Randomization

The participants were assigned into CBT-based intervention group, sexual health education receiving group, and control group with a ratio of 1:1:1 by block randomization using Random Allocation Software (RAS) with a block size of 6 and 9. The type of allocation was written on paper and put in sequentially numbered opaque envelopes for the allocation concealment. A non-involved person in the sampling opened the envelopes consecutively. The outcome assessor (the fifth author) was blinded.

### Intervention

One of the intervention groups received CBT based on the ABCDE model during eight 60–90 min sessions once a week by the third author under the supervision of the fourth author (psychologist). The basic idea behind the ABCDE model is that our emotions and behaviors (C: Consequences) are not directly determined by activating events (A) but rather by the way these events are cognitively processed and evaluated (B: Beliefs). The disputation (D) of the irrational belief into a rational belief results in new effects (E) [26]. The minimum and maximum number of participants in each session was 5 and 7, respectively (Table 1). The other intervention group received 5–7 sessions of sexual health education for 60–90 min, including training and explaining the structure and function of the reproductive system and the stages of the sexual response cycle, familiarizing women with their sexual and reproductive rights, providing examples of sexual violence against women and how to protect themselves, explaining the sexually transmitted infections and their prevention methods, genital hygiene, and healthy and responsible sexual behaviors, and training sexual and communication skills. The third author (sexologist) conducted five educational group sessions and two individual sessions on a per-need basis. The control group did not receive any intervention during the study. Eight weeks after the intervention, members of all three groups filled out the Hulbert sexual assertiveness index and Larson sexual satisfaction questionnaire. In line with ethical considerations, sexual health education sessions were held for the control group after the completion of the research and data analysis.

**Table 1:** Summary of counseling sessions for CBT group (*n* = 22)

Sessions	Objectives	Content of sessions
First	Identifying incompatible thoughts and beliefs, challenging incompatible thoughts and beliefs	Greetings and introducing, stating the objectives and logic of cognitive-behavioral therapy, communicating and collecting background information, introducing the cognitive-behavioral approach, brief training on the anatomy and physiology of the male and female reproductive system, and training on the sexual physiology
Second		Expressing the importance of sexual assertiveness in women, examining the causes of the lack of sexual expression and desire in women, and counseling women to identify the reasons for their lack of sexual assertiveness, training the need for self-expression and its benefits in life, investigating women’s sexual preferences and desires and how expressing them to their husbands, training on writing their sexual needs and desires to their partner, and providing solutions to further increase sexual expression Homework: Participants should write their expected changes from the counseling plan as homework for the next session
Third		Reviewing the previous session, examining the causes of low sexual self-efficacy, identifying the person’s dysfunctional beliefs and unconscious thoughts about her ability in sexual relations, evaluating dominant negative sexual attitudes, introducing cognitive distortions, and assessing illogical sexual beliefs and explaining them, overviewing the sexual attitudes of women with low sexual self-efficacy Homework: Revising the cognitive distortions, practicing the identification of the cases of cognitive distortions and dysfunctional beliefs using the thought recording sheet
Fourth		Reviewing the previous session, examining the coping strategies with cognitive distortions, reconstructing maladaptive self-talks, and resolving misunderstandings (cognitive skills), training to increase positive self-talks, and training problem-solving skill and its role in reducing factors related to marital conflicts
Fifth		Determining the boundaries related to sexual activity, what an individual expects from herself and her partner in a sexual relationship, training the cycle of sexual response, training the technique of self-arousal, training non-sexual sensation, prohibiting sexual relationship until the end of body sensation training, changing negative attitudes towards sexual issues, and cognitive reconstruction of dysfunctional sexual thoughts, and introducing the ABCDE model Homework: Write your sexual behaviors based on the ABC model, and paying attention to each other’s sexual needs
Sixth		Reviewing the previous session, training on how to reach orgasm at the same time based on the sensitive points of the couple, training on sexual fantasy, removing the ban on touching genitals, training on types of sexual intercourse methods, and training relaxation and Kegel exercises Homework: Relaxation exercise and Kegel exercise
Seventh		Describing personality differences, negotiating and agreeing with husband on creating common strategies, training on how to express the emotions of couples to each other, and increasing the verbal intimacy of couples and positive interactions Homework: Exercise to deal with the return of previous behavior and attitudes
Eighth		Summarizing the contents and evaluating the different techniques used by the participants, giving feedback about the effectiveness or ineffectiveness of the counseling, solving the existing problems, and evaluating the positive results of the intervention and the level of individuals’ satisfaction with the counseling

### Data collection tools

The data were collected using the demographic and obstetric characteristics, Hulbert sexual assertiveness index, and Larson sexual satisfaction questionnaire.

### Demographic and obstetric characteristics

The demographic and obstetric characteristics included the variables of age, education, occupation, family income, number of intercourses per month, contraception method, length of marriage, etc.

### Hulbert sexual assertiveness index

Hulbert sexual assertiveness index, developed by Hulbert, was used to measure women’s sexual assertiveness. This 25-item tool is scored on a 5-point Likert scale, ranging from always (0) to never (4). Items 3, 4, 5, 7, 12, 15, 16, 17, 18, 21, 22, and 23 are scored inversely (always = 4 to never = 0). The score range is from 0—100, as 0 -33 indicates a low level of sexual assertiveness, 33–50 represents moderate sexual assertiveness, and a score above 50 illustrates a high level of sexual assertiveness. The test–retest reliability and internal consistency coefficient of the instrument were obtained 0.86 and 0.89, respectively [27]. In the Iranian version of the instrument, the Cronbach's alpha coefficient and Intraclass Correlation Coefficient (ICC) were reported 0.92 and 0.91, respectively [28].

### Larson sexual satisfaction questionnaire

The sexual satisfaction was assessed using Larson sexual satisfaction questionnaire, developed by Larson et al. [29]. The questionnaire consists of 25 items and 4 dimensions, including libido, sexual attitude, quality of sexual life, and sexual adjustment. Each item is scored on a 5-point Likert scale, ranging from never = 1 to always = 5. Items 4—9, 11, 14, 15, 18, 20, 24, and 25 are inversely scored from 5 to 1, respectively. The minimum possible score is 25 and the maximum is 125, as a score less than 50 reflects lack of sexual satisfaction, 51–75 indicates low satisfaction, 76–100 displays moderate satisfaction, and more than 100 demonstrates high sexual satisfaction. In the Persian version of the instrument, the Cronbach's alpha coefficient and ICC were reported as 0.9 and 0.86, respectively [30].

### Data analysis

The data were analyzed using SPSS_25_ software and Shapiro–Wilk test was employed to assess the data normality. The mean score of sexual assertiveness and sexual satisfaction was compared among the three groups using ANOVA test before the intervention and ANCOVA test after the intervention by controlling the effect of the baseline score.

## Results

**Fig. 1:**
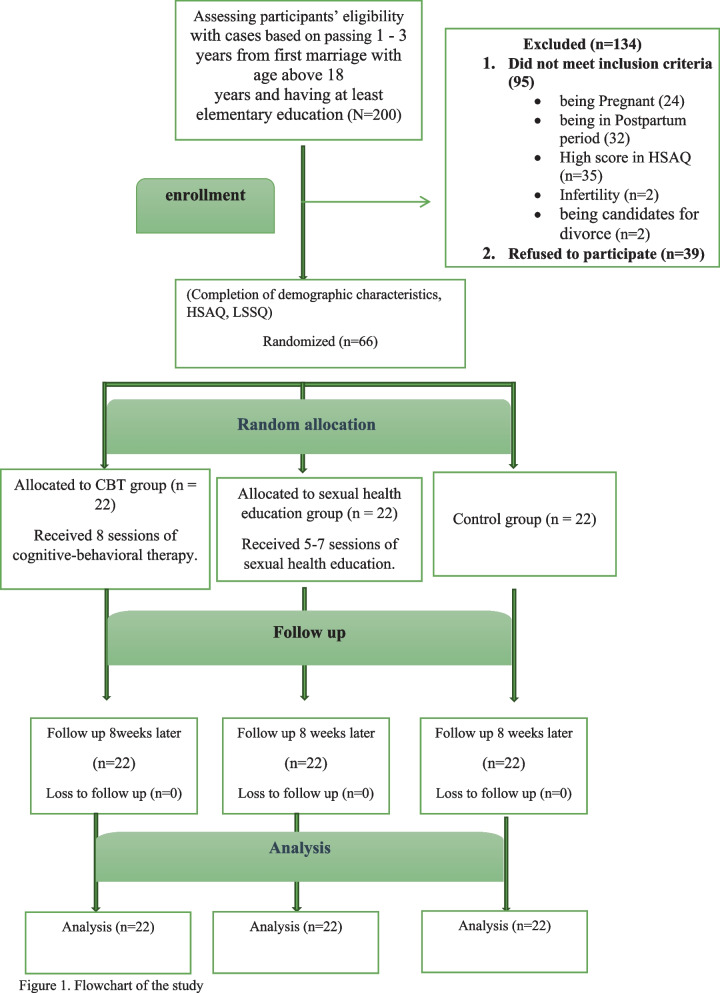
Flowchart of the study

This clinical trial was conducted to evaluate the effect of sexual health education and CBT on the sexual assertiveness and satisfaction of newly married women from June to March 2021. Of 200 newly married women, 66 eligible women were randomly assigned to three groups (*n* = 22 in each group). None of the participants was excluded from the study and the data of all participants were analyzed (Fig. 1).

The mean (SD) age of subjects was 26.18 (2.93) in the CBT group, 26.31 (2.81) in the sexual health education group, and 27.13 (3.07) in the control group. No significant difference in mean age score was found among groups using ANOVA (*P* = 0.51). The level of education of the majority of women in all three groups was academic (68.1% in the CBT group, 90.9% in the sexual health education group, and 86.3% in the control group). There was no significant difference in education among groups using the chi-square test (*P* = 0.05). The average length of marriage (year) was 2.59 in the CBT group, 2.54 in the sexual health education group, and 2.04 in the control group (*P* = 0.10). The mean (SD) number of intercourses per week was 2.45 (0.85) in the CBT group, 2.40 (1.05) in the sexual health education group, and 2 (0.81) in the control group (*P* = 0.20). In general, there was no statistically significant difference in the demographic and obstetric characteristics of participants among the three groups (Table 2).

**Table 2:** The demographic and obstetric characteristics of the participants

**Variable**	**Cognitive behavioral therapy group** **(*n* = 22)** **N(%)**	**Sexual health education group** **(*n* = 22)** **N(%)**	**control group** **(*n* = 22)** **N(%)**	***P*-value**
**Age**^a^	26.18 (2.93)	26.31 (2.81)	27.13 (3.07)	0.512*
**Spouse age**^a^	31.45 (3.29)	31.18 (2.78)	31.59 (3.17)	0.905*
**Marriage duration**^a^	2.59 (0.59)	2.54 (0.5)	2.04 (0.78)	0.153*
**Coitus frequency/week** ^a^	2.45 (0.85)	2.40 (1.05)	2 (0.81)	0.201*
**Level of Education**				0.054**
Elementary/ guidance	0 (0)	0 (0)	0 (0)	
High school Diploma	7 (31.8)	2 (9.1)	3 (13.6)	
Academic	15 (68.1)	20 (90.9)	19 (86.3)	
**Spouse level of education**				0.408**
Illiterate	1 (4.5)	0 (0)	0 (0)	
Elementary/ guidance	1 (4.5)	0 (0)	2 (9.1)	
High school/ diploma	7 (31.8)	5 (22.7)	7 (31.8)	
Academic	13 (59.1)	17 (77.3)	13 (59.1)	
**Occupation**				0.07**
Housekeeper	15 (68.2)	11 (50)	9 (40.9)	
Employed	7 (31.8)	11 (50)	13 (59.0)	
**Household income level**				0.995**
Enough	2 (9.1)	2 (9.1)	2 (9.1)	
Somewhat enough	15 (68.2)	16 (72.7)	16 (72.7)	
Inadequate/Not enough	5 (22.7)	4 (18.2)	4 (18.2)	
**Contraception method**				0.583**
Withdrawal	10 (45.5)	12 (54.5)	8 (36.4)	
Condom	8 (36.4)	5 (22.7)	8 (36.4)	
Pills/ injection	0 (0)	2 (9.1)	1 (4.5)	
No method	4 (18.2)	3 (13.6)	5 (22.7)	

^a^mean(SD)

^*^ANOVA

^**^Chi-square

The mean (SD) score of the sexual assertiveness in the CBT group enhanced from 48.77 (13.94) before the intervention to 69.37 (7.28) after the intervention. The mean (SD) score of the sexual assertiveness in the sexual health education group increased from 48.9 (11.39) before the intervention to 66. 94 (7.42) after the intervention and changed from 45.04 (15.87) before the intervention to 42.74 (14.11) after the intervention in the control group. Before intervention, there was no significant difference among three groups, using ANOVA test (*P* = 0.57), and also in comparison with each other, using independent t-test. Eight weeks after the intervention, the mean score of sexual assertiveness in the CBT group was significantly more than that in the control group, using ANCOVA while controlling the baseline scores [AMD: 26.62, 95% CI: 20.38 to 32.87, *P* < 0.001]. Further, the mean score of the sexual assertiveness in the sexual health education group was significantly more than that in the control group [AMD: 24.19, 95% CI: 17.93 to 30.44, *P* < 0.001], However, there was no statistically significant difference between the two intervention groups [AMD: 2.43, 95% CI: -3.65 to 8.52, *P* = 0.69] (Table 3).

**Table 3:** The Comparison of sexual assertiveness before and after intervention between three groups

**Group**	**Before intervention** **Mean (SD)**		**After intervention** **Mean (SD)**	
**Cognitive behavioral therapy**	48.77 (13.94)		69.37 (7.28)	
**Sexual health education**	48.90 (11.39)		66.94 (7.42)	
**Control**	45.04 (15.87)		42.74 (14.11)	
***P* value**	0.579^a^		< 0.001**	
**Group comparison**	**Mean Difference (95%Confidence interval)**	*P* value***	**Mean Difference (95%Confidence interval)**	*P* value**
**CBT with control group**	3.72 (-5.36 to 12.81)	0.358	26.62 (20.38 to 32.87)	< 0.001
**Sexual health education with control group**	3.86 (-4.57 to 12.27)	0.06	24.19 (17.93 to 30.44)	< 0.001
**CBT with Sexual health education group**	-0.13 (-7.88 to 7.61)	0.368	2.43 (-3.65 to 8.52)	0.699

^a^ANOVA

^**^ANCOVA

^***^ Independent t-test

The mean (SD) score of the sexual satisfaction in the CBT group enhanced from 73.13 (13.53) before the intervention to 86.57 (7.5) after the intervention. The mean (SD) score of the sexual satisfaction in the sexual health education group increased from 74.95 (8.30) before the intervention to 84.93 (6.34) after the intervention and changed from 69.04 (10.75) before the intervention to 66.44 (10.11) after the intervention in the control group. Before intervention, a significant difference was not observed among three groups, using ANOVA test (*P* = 0.20), and also in comparison with each other, using independent t-test. Eight weeks after the intervention, the mean difference of sexual satisfaction between the CBT and control groups was 20.12 (95% CI: 16.23 to 24.02, *P* < 0.001). In addition, the mean difference of sexual satisfaction between the sexual health education and control groups was 17.94 (95% CI: 14.05 to 21.84, *P* < 0.001). However, there was no statistically significant difference between the two intervention groups [AMD: 2.18, 95% CI: -1.54 to 5.91, *P* = 0.39] (Table 4).

**Table 4:** The Comparison of sexual satisfaction before and after intervention between three groups

**Group**	**Before intervention** **Mean (SD)**		**After intervention** **Mean (SD)**	
**Cognitive behavioral therapy**	73.13 (13.53)		86.57 (7.5)	
**Sexual health education**	74.95 (8.30)		84.93 (6.34)	
**Control**	69.04 (10.75)		66.44 (10.11)	
***P* value**	0.202^a^		< 0.001**	
**Group comparison**	**Mean Difference (95%Confidence interval)**	*P* value***	**Mean Difference (95%Confidence interval)**	*P* value**
**CBT with control group**	4.09 (-3.36 to 11.54)	0.535	20.12 (16.23 to 24.02)	< 0.001
**Sexual health education with control group**	5.90 (-2.28 to 14.09)	0.225	17.94 (14.05 to 21.84)	< 0.001
**CBT with Sexual health education group**	-1.81 (-10 to 6.37)	0.930	2.18 (-1.54 to 5.91)	0.399

^a^ANOVA

^**^ANCOVA

^***^Independent t-test

## Discussion

The results of the present study indicated that the sexual assertiveness of women in both intervention groups increased significantly compared to the control group. In line with the findings of the present study, the study results of Chizary et al. [22] demonstrated a significant improvement in orgasm, sexual assertiveness, and sexual function of women with secondary orgasmic dysfunction in the intervention group compared to those in the control group after eight sessions of CBT. In addition, Kristanti et al. [31] reported a significant improvement in the sexual assertiveness of women with low libido after five sessions of CBT. In another study on women with low knowledge and intimacy scores, Salimi and Fatehizadeh [32] showed a significant improvement in the sexual assertiveness in the intervention group compared to the control group after 6-session of sexual education. In a study conducted by Weinhardt et al., a significant increase in the sexual assertiveness of women who were at high risk for HIV was reported after 10-session of CBT [23].

Based on the findings of the present study, following the improvement of sexual assertiveness, low sexual satisfaction of newly married women altered to moderate satisfaction in both intervention groups. In line with the results of the present study, the findings of the previous studies confirmed the relationship between the sexual assertiveness and sexual satisfaction [4, 22].

The results of the present study illustrated that following CBT-based counseling and sexual health education, the mean score of sexual satisfaction of both intervention groups increased significantly compared to that of the control group. In the same vein, Omidvar et al. [33] found that both mindfulness-based counseling and CBT were effective in increasing the sexual satisfaction of women with vaginismus disorder in comparison with control group. Furthermore, in a study by Morvari et al., [34] after 8-session of CBT, the sexual satisfaction of women with breast cancer after mastectomy increased significantly. Additionally, Marvi et al. [35] indicated a significant improvement in the sexual function and sexual satisfaction of infertile women after three sessions of sexual education based on sexual health model. In other study, following six group sessions of sexual education, a significant increase in the sexual satisfaction was observed in women with low sexual satisfaction [36].

Consistent with the results of the present study, in the study of Bokaie et al. [37] on primiparous women, sexual satisfaction significantly increased compared to control group after 8-session of cognitive behavioral therapy.

Inconsistent with the findings of this research, the study results of Vakilian et al. [38] after 7-session of CBT for pregnant women with low sexual function score, demonstrated no significant difference in the sexual function and sexual satisfaction of the women in the intervention group compared to those in the control group one and three months after the intervention, which is probably due to the different study population and tools used. In addition, Wannakosit et al. [39] following a session of sexual education during pregnancy, reported no significant difference in the sexual behaviors and sexual satisfaction of women in the intervention group compared to those in the control group, twelve weeks after the intervention. The reason for the contradictory results can be attributed to the few numbers of training sessions and the different population of the study in the aforementioned study.

### Strengths and limitations

Designing the study based on clinical trials principles, such as random allocation and allocation concealment to eliminate the selection bias in addition to having no attrition rate were among the strengths of this study. There are some limitations in this research, including self-reporting of data, impossibility of blinding the participants due to the nature of the study, lack of long-term follow-up, and failure to investigate the spouses, due to the taboo of sexual issues in Iran and their unwillingness to participate in the study. Moreover, the baseline mean score of sexual assertiveness and satisfaction was very close to the cut-off limit in all groups. The findings may be different, if the studied population had lower baseline assertiveness and satisfaction. Therefore, it is suggested to perform further studies in other population.

## Conclusion

The results of the present study revealed that CBT and sexual health education are effective in improving newly married women’s sexual assertiveness and satisfaction. Considering that sexual health education compared to CBT does not require complex counseling skills and can be easily provided by women’s health care providers in health centers during a few sessions, it can be used as a preferred approach in promoting sexual assertiveness and satisfaction of newly married women with low sexual expression power.

Any procedure which rises awareness about one’s sexual life contains the potential to promote changes in sexual behaviour and it’s outcomes. Thus, further studies need to evaluate the effectiveness of different interventions on sexual satisfaction in women with sexual dysfunction.

## Data Availability

The data sets used and/or analyzed during the present study are available from the corresponding author on reasonable request.

## Abbreviations

CBT: Cognitive-Behavioral Therapy
SD: Standard Deviation
IRCT: Iranian Registry of Clinical Trials
HIV: Human Immunodeficiency Virus
RAS: Random Allocation Software
ICC: Intraclass Correlation Coefficient
AMD: Adjusted Mean Difference
CI: Confidence Interval
